# Neuroimaging and immunofluorescence of the *Pseudopus apodus* brain: unraveling its structural complexity

**DOI:** 10.1007/s00429-025-02940-6

**Published:** 2025-05-28

**Authors:** S. Jiménez, R. Morona, M. J. Ruiz-Fernández, E. Fernández-Valle, D. Castejón, M. I. García-Real, J. González-Soriano, N. Moreno

**Affiliations:** 1 Achucarro Basque Center for Neuroscience, Scientific Park of the University of the Basque Country (UPV/EHU), 48940 Leioa, Spain; 2 Department of Cell Biology, Faculty of Biological Sciences, Complutense University, Avenida José Antonio Nováis 12, 28040 Madrid, Spain; 3 DB Diagnóstico Por Imagen Veterinario, Calle Tordesillas 4, 28925 Alcorcón, Madrid Spain; 4 ICTS Bioimagen Complutense, Complutense University, Paseo de Juan XXIII 1, 28040 Madrid, Spain; 5 Department of Animal Medicine and Surgery, Faculty of Veterinary, Complutense University, Avenida Puerta de Hierro s/n, 28040 Madrid, Spain; 6 Department Section of Anatomy and Embryology, Faculty of Veterinary, Complutense University, Avenida Puerta de Hierro s/n, 28040 Madrid, Spain

**Keywords:** Ophisaurus, Reptiles, Magnetic resonance imaging (MRI), Brain, Atlas

## Abstract

The present study provides an in-depth neuroanatomical characterization of the brain of *Pseudopus apodus*, combining magnetic resonance imaging (MRI) with histological analysis by immunofluorescence. In the telencephalon, the pallial regions showed distinct anatomical features, including a cortical structure, a dorsal ventricular ridge and the spherical nucleus, but prominent layering patterns, observable on histological slides, were not fully resolved by MRI. Subpallial structures, such as the nucleus accumbens and the basal ganglia, were delineated with histological clarity and further supported by MRI. In the hypothalamic and diencephalic regions, the dense and complex cellular composition made precise delineation of individual nuclei difficult by MRI, in contrast to the histological accuracy, however by MRI the identification of the major tracts running through these domains are clearly identifiable. Mesencephalic and rhombencephalic structures, including the optic tectum, isthmic nuclei, cerebellum, and reticular groups, were systematically described using a combination of histological and MRI techniques. In addition, immunofluorescence analysis of specific markers, such as Calretinin, ChAT, Isl1, Satb1, Serotonin and Tyrosine Hydroxylase, provided higher resolution of functional sub-regions, allowing precise identification of boundaries and facilitating comprehensive regional mapping, showing complex organizational arrangements, both in rostral regions, such as the dorsal ventricular crest, and in caudal regions, within the tegmental and posterior nuclei of the brain, including the ventral tegmental area, substantia nigra and raphe nuclei. These findings establish a robust neuroanatomical framework for *Pseudopus apodus*, contributing significantly to the understanding of reptile brain organization and providing valuable insights into the evolutionary adaptations underlying a limbless lizard neuroanatomy.

## Introduction

Magnetic resonance imaging (MRI) is a very powerful and widely used technique in medical studies, relatively incipient in fields such as evolutionary neurobiology or paleobiology, although in recent years interest has grown due to its wide applicability as a non-invasive technique permitting the study of models that would otherwise be inaccessible (Vickery et al. 2020; Oelschläger et al. 2007; Mietchen et al. 2007; Hoffmann et al. 2014). And thus, recently there have been significant advances in MRI studies in animals not traditionally used in neuroscience research. In particular, an increasing number of brain atlas of reptile species have been published in recent years (Hoops et al. 2018, 2021; Pritz et al. 2020; Behroozi et al. 2018; Jiménez et al. 2024).

Among reptiles, and specifically lizards, the family Anguidae is commonly known as glass lizards or glass snakes, as it includes species that are often limbless, but anatomically they can be identified as lizards by the characteristic shape of their heads, the presence of eyelids and the external openings of their ears. It comprises approximately 20 recognized species, originating in North America, dispersing to Europe and spreading to Asia during the Oligocene (Macey et al. 1999). In particular, the European glass lizard, *Pseudopus apodus* (Pallas 1775), is the largest legless lizard in Europe (Arnold and Ovenden 2002; Obst 1981), and the only extant specie with a distribution spanning Asia Minor, Central Asia, southeastern Europe, and the Balkans (Jandzik et al. 2018). Fossil evidence indicates that *Pseudopus apodus* has been present in the region since the early Miocene (Klembara 2015). Molecular analyses have confirmed the existence of two subspecies (Obst 1978, 1981), closely related to North American anguines such as *Ophisaurus*, but it forms a monophyletic clade with the genus *Anguis* (Macey et al. 1999; Pyron et al. 2013).

Research on the brain of *Pseudopus apodus* is almost non-existent, but there are studies on the closely related genus Ophisaurus (Kulikov and Safarov 1969; Ivazov and Belekhova 1982; Ivazov 1983; Belekhova and Ivazov 1983; Belekhova and Nemova 1987, 1988; Belekhova 1990, 1991; Pierre et al. 1990; Rio et al. 1992) and with which many comparisons can be inferred, suggesting that its neuroanatomical organization is most likely related to its ecological role as a generalist diurnal terrestrial predator (Rifai et al. 2005), highly dependent on sensory information for navigation and foraging. *Pseudopus apodus* shows diverse habitat preferences depending on its geographic range and, although sexual dimorphism is not prominent, it has been observed in certain populations (Çiçek et al. 2014; Kukushkin and Dovgal 2018). Therefore, the brain of *Pseudopus apodus* consists of the forebrain, involved in olfactory processing, spatial navigation and social behavior; the midbrain, for visual and auditory processing, crucial for its diurnal lifestyle; and the hindbrain, which controls basic motor and autonomic functions and coordinates the undulating locomotion necessary for terrestrial movement.

Noticeably, the lack of in-depth neuroanatomical studies and MRI techniques represent an important gap in our knowledge of this species, especially considering the ecological data available. Comparison of these data with neuroanatomical information would provide valuable insights into anatomical-functional relationships and its evolutionary position within reptiles, particularly considering that it is a legless lizard. Therefore, the main objective of the present study is to provide a detailed analysis of the brain of this model, through the combined use of MRI and immunofluorescence identification of conserved markers, complementing the recent study on its anatomy (García-Real et al. 2025) and following in the wake of previous neuroanatomical data recently published in reptiles (Jiménez et al. 2024). Thus, they contribute to complete this important framework of information for understanding this evolutionary node.

## Materials and methods

### Animals and tissue preparation

For the present study, we used 4 healthy adult *Pseudopus apodus* individuals (2 males and 2 females), homogeneous in size (with an average size of 100 cm) between 5 and 10 years of age. The regulations and laws established by European Union (2010/63/EU) and Spain (Royal Decree 118/2021) for care and handling of animals in research has been followed and the approval from the Ethic committee of the Complutense University (O.H. (CEA)- UCM-NP0409032022-2022).

By intraperitoneal injection (sodium pentobarbital; 50–100 mg/kg, Normon Labs, Madrid, Spain) the animals were anesthetized and perfused transcardially (4% paraformaldehyde in a 0.1 M phosphate buffer, pH 7.4) and the brains removed from the skull.

### MRI ex vivo

MRI studies were performed in two brains at BioImaC (ICTS BioImagen Complutense, Madrid, Spain), node of the ICTS ReDIB (https://www.redib.net/). A 4.7-teslas MRI scanner [Biospec 47/40; Bruker BioSpin GmbH, Ettlingen, Germany], equipped with a 6-cm gradient system that provides a gradient strength of 900 mT/m, was used for ex vivo studies. The brains were drained and immersed in a proton-free susceptibility-matching fluid, Fluorinert® FC‐40 (Sigma‐Aldrich, Saint Louis, MO, USA) and placed inside a 3.5-cm volume radiofrequency coil. The MRI experiment consisted of three-dimensional T2 horizontal weighted images (T2 WI) used for identification of brain structures. Three-dimensional T2 WI were obtained using a rapid acquisition with relaxation enhancement (RARE) sequence, with a repetition time (TR) = 2622 s, echo train length = 4, interecho interval = 35 ms (resulting in an effective echo time (TE) = 82 ms), number of averages = 4, field of view (FOV) = 30 × 7.5 × 7.5 mm3. The acquired matrix size was 384 × 96 × 96. The raw data were zero-filled to get a reconstructed matrix size of 512 × 128 × 128 (resolution in each direction 59 μm) and the total acquisition time ~ 6 h 42 min. Then horizontal images were reconstructed in axial, sagittal and coronal orientations with an isotropic resolution of 25 μm.

### Histological and immunolabeling experiments and controls

Two brains were subjected to the same experimental procedure of MRI followed by histological analysis. The other two brains were directly processed to immunohistochemistry.

For Nissl staining, brain sections were immersed in cresyl violet, followed by differentiation and dehydration with alcohol for optimal visualization of neuronal nuclei. Additionally, we performed immunohistochemistry (refer to Table 1 for commercial specifications, immunogen details, and antibody dilutions) for single and combined detection of Calretinin (CR), Choline Acetyltransferase (ChaT), Islet-1 (Isl1), Special AT-rich sequence-binding protein 1 (Satb1), Serotonin (5-HT) and Tyrosine Hydroxylase (TH).

**Table 1:** List of primary and secondary antibodies used, immunogen, commercial supplier and dilution

Name	Immunogen	Commercial supplier	Dilution
CB	E-coli-produced recombinant rat calbindin D-28 k	Polyclonal rabbit anti-calbindin D-28; Swant, Bellinzona, Switzerland. Catalog No. CB-38a	1:500
ChaT	Developmental Studies Hybridoma Bank, mouse monoclonal, Cat# 40.2D6	Mouse monoclonal, Developmental Studies Hybridoma Bank. Catalog No. 40.2D6	1:100
CR	E-coli-produced recombinant human calretinin	Polyclonal rabbit anti-calretinin; Swant, Bellinzona, Switzer- land. Catalog No. 7699/4	1:1000
Isl1	Amino acids 247–349 at the C-terminus of rat Islet 1	Mouse monoclonal, Developmental Studies Hybridoma Bank. Catalog No. 40.2D6	1:500
Satb1	Amino acids 241–310 located in the internal region of human-origin SATB1	Monoclonal mouse, Santa Cruz. Catalog No. sc-376096	1:100
Ser (5-HT)	Serotonin coupled to BSA with paraformaldehyde	Polyclonal rabbit anti-5-HT, Immunostar. Catalog No: 20080	1:1000
TH	TH purified from rat pheochromocytoma cells	Mouse monoclonal, ImmunoStar. Catalog No. P22941	1:1000

Immunofluorescence co-labeling was conducted on free-floating sections obtained with a freezing microtome (30–40 μm thickness) in the transverse or sagittal planes. The procedure was as follows: (1) Primary antibody incubation was carried out for 48 h at 4 °C (refer to Table 1 for antibody specifics); (2) Secondary antibody incubation, based on the species of the primary antibody, was performed for 90 min at room temperature using a 1:500 dilution. The secondary antibodies used were Alexa 594-conjugated goat anti-rabbit (red fluorescence; Molecular Probes, Eugene, OR; catalog #A-11037) and Alexa 488-conjugated goat anti-mouse (green fluorescence; Molecular Probes; catalog #A-21042). Following incubation, the sections were mounted on glass slides and coverslipped with fluorescence mounting medium containing 1.5 µg/ml 4′,6-diamidino-2-phenylindole (DAPI) for DNA counterstaining (Santa Cruz; catalog #SC-24941).

Control experiments for the immunohistochemical procedures included the omission of either the primary or secondary antibody, as well as incubating selected sections with preimmune mouse or rabbit serum instead of of the primary antibody. No residual staining was observed in any control section. In addition, previous studies analyzing the specificity of the antibodies used have shown that they exhibit specificity in the reptile species tested and/or comparable and coherent expression patterns (Medina et al. 1994; Guirado et al. 1999b; Morona et al. 2006; Moreno et al. 2010, 2012; Jiménez et al. 2025).

### Analysis of photomicrographies

The sections were analyzed with an Olympus BX51 microscope equipped for fluorescence and photographed with a digital camera (Olympus DP74). Contrast and brightness of the photomicrographs were adjusted in Adobe Photoshop CS6 (Adobe Systems, San Jose, CA) and figures were mounted in Canvas X (ACD Systems, Canada).

## Results

**Fig. 1:**
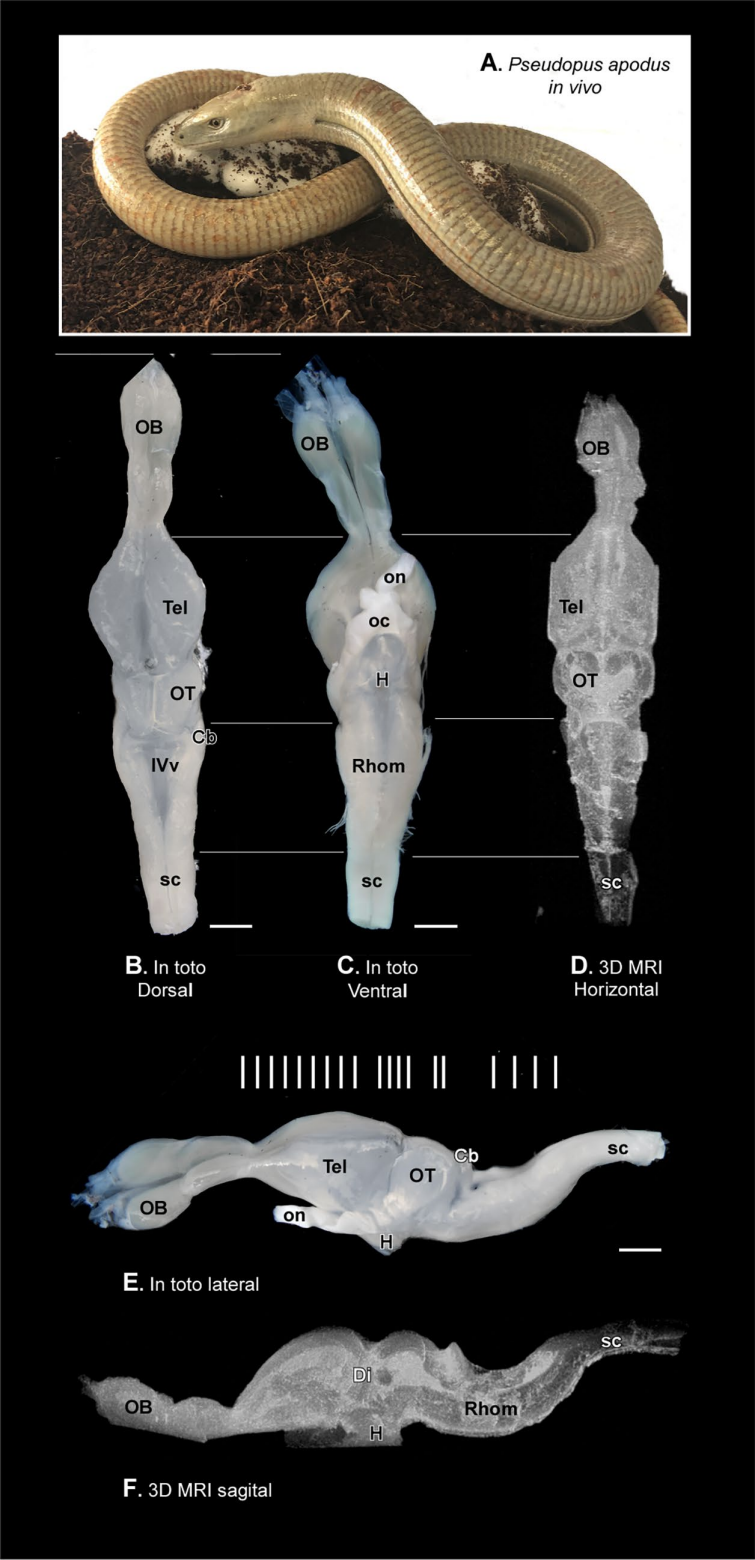
*Pseudopus apodus* images: in vivo photograph and anatomical views of the brain through 3D MRI reconstruction. **A** In vivo photograph of *Pseudopus apodus*. **B** Dorsal view, **C** ventral view, and **E** lateral view of the brain of *Pseudopus apodus*. **D**, **F** The 3D MRI reconstruction in dorsal and sagittal views, respectively. Scale bar = 1 mm. See the list of abbreviations for further reference

**Fig. 2:**
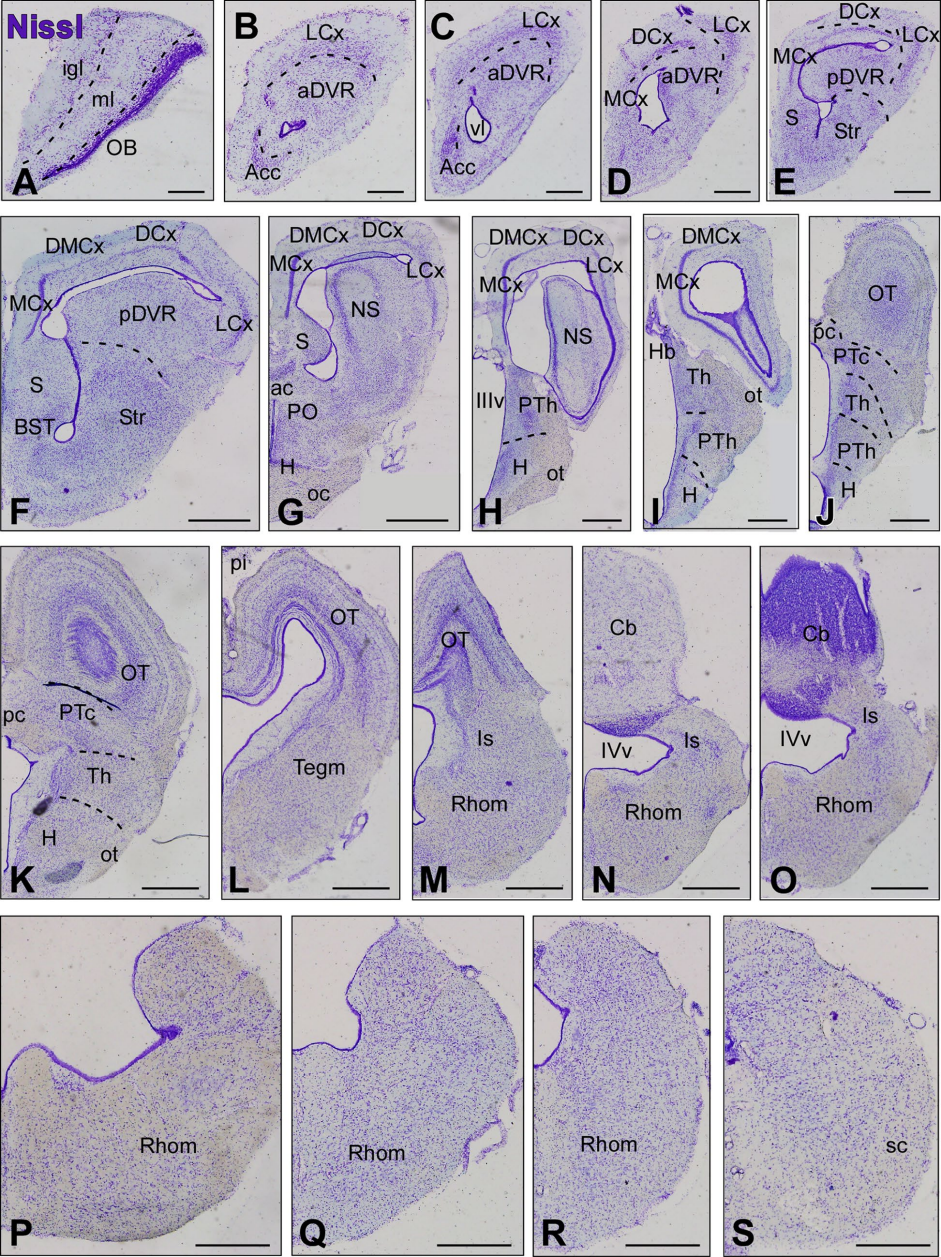
Nissl-stained sections of the *Pseudopus apodus* brain. Transversal Nissl-stained sections of the brain of *Pseudopus apodus*, from the rostral olfactory bulb (A) to the rostral spinal cord (S). Scale bar = 500 μm. See the list of abbreviations for reference

**Fig. 3:**
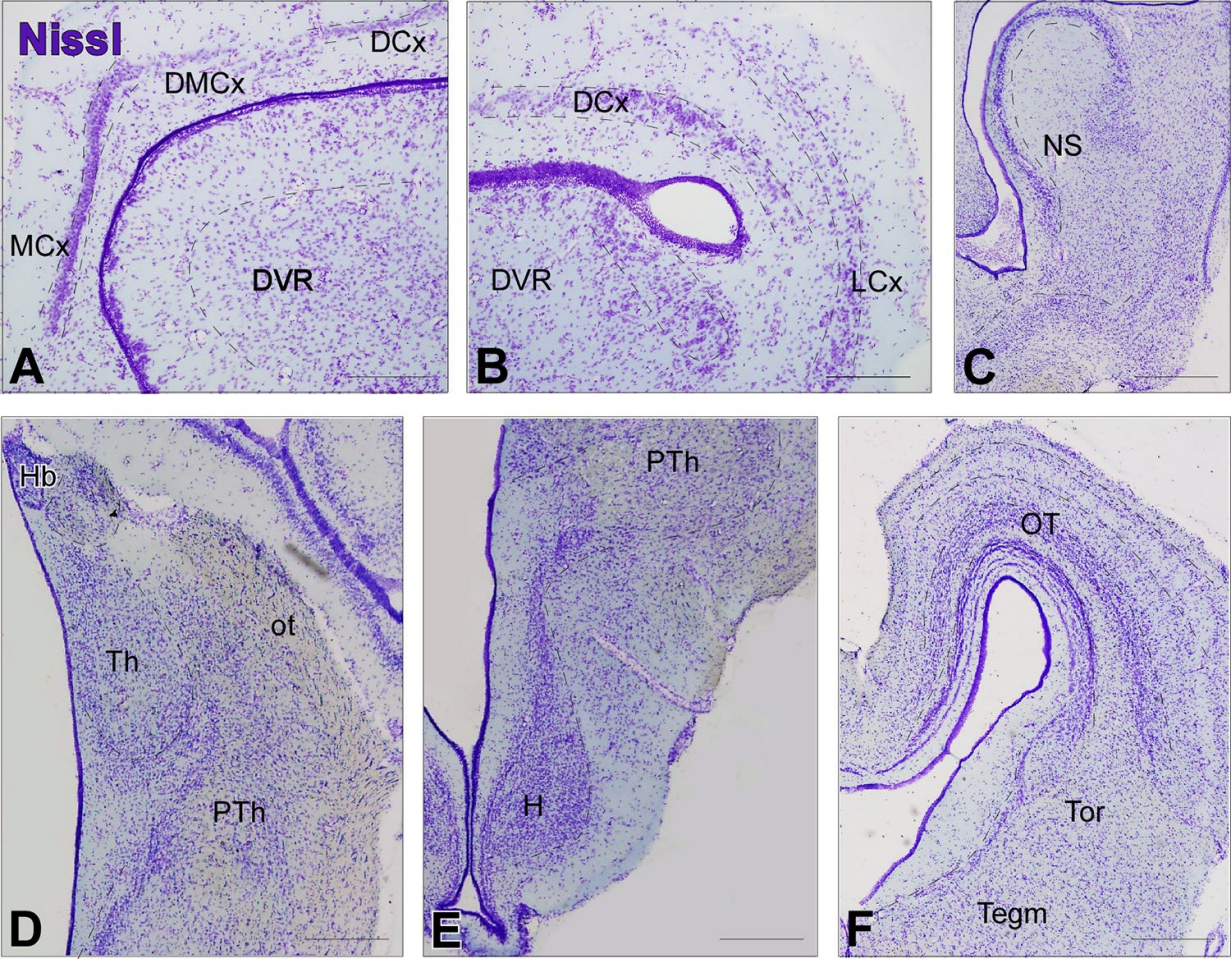
Enlarged view of specific regions highlighted in Fig. 2. Transverse Nissl-stained brain sections of *Pseudopus apodus* showing detailed views of the pallial (**A**–**C**), thalamic (**D**), hypothalamic (**E**), and tectal (**F**) regions. Scale bar = 200 μm. See the list of abbreviations for reference

**Fig. 4:**
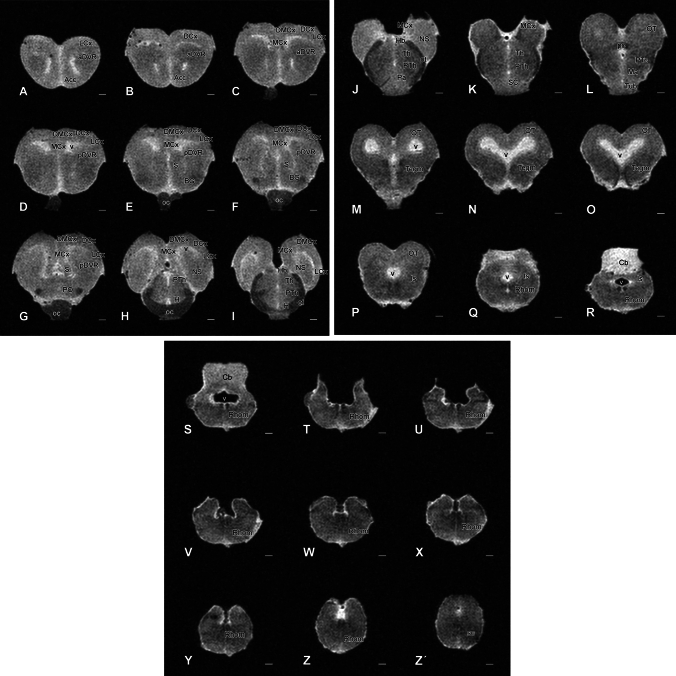
Rostral-caudal transversal sections of the *Pseudopus apodus* brain from 3D MRI. Transversal sections of the *Pseudopus apodus* brain, organized rostrocaudally, with the main brain regions indicated. Scale bar = 500 μm. See the list of abbreviations for reference

Figure 1 illustrates the appearance of the *Pseudopus apodus* study model (Fig. 1A), along with dorsal (Fig. 1B), ventral (Fig. 1C), and lateral (Fig. 1E) views of the intact brain, as well as the three-dimensional reconstruction of the brain following MRI scans in the dorsal (Fig. 1D) and sagittal (Fig. 1F) planes. The white lines in Fig. 1E show the approximate section levels observed in the transverse slices presented in Figs. 2, 3 and 4. Transverse sections depicting the rostrocaudal organization of the adult *P. apodus* brain, stained with Nissl for nuclear visualization, are shown in Figs. 2 and 3. Corresponding rostrocaudal transverse MRI images were selected at similar levels (Fig. 4). The same assay has also been visualized in sagittal and dorsal planes (Fig. 4). Finally, for the specific identification of nuclei and regions in the *P. apodus* brain, the expression of specific markers (see Table 1) has been analyzed (Figs. 6, 7, 8 and 9). The anatomical description will be conducted in a topographical rostrocaudal sequence just for a ready comparison with previous studies and following previous maps and descriptions of reptiles (specifically of lizards), due to the limited number of studies on the brain of this species. This is remarkable in the description of the telencephalon, but the interpretation of the results is always considered within the prosomeric paradigm. Furthermore, the discrepancies observed in any region or nuclei, in comparison to what has been described in reptiles in general, or in lizards in particular, will be commented in the discussion section.

**Fig. 5:**
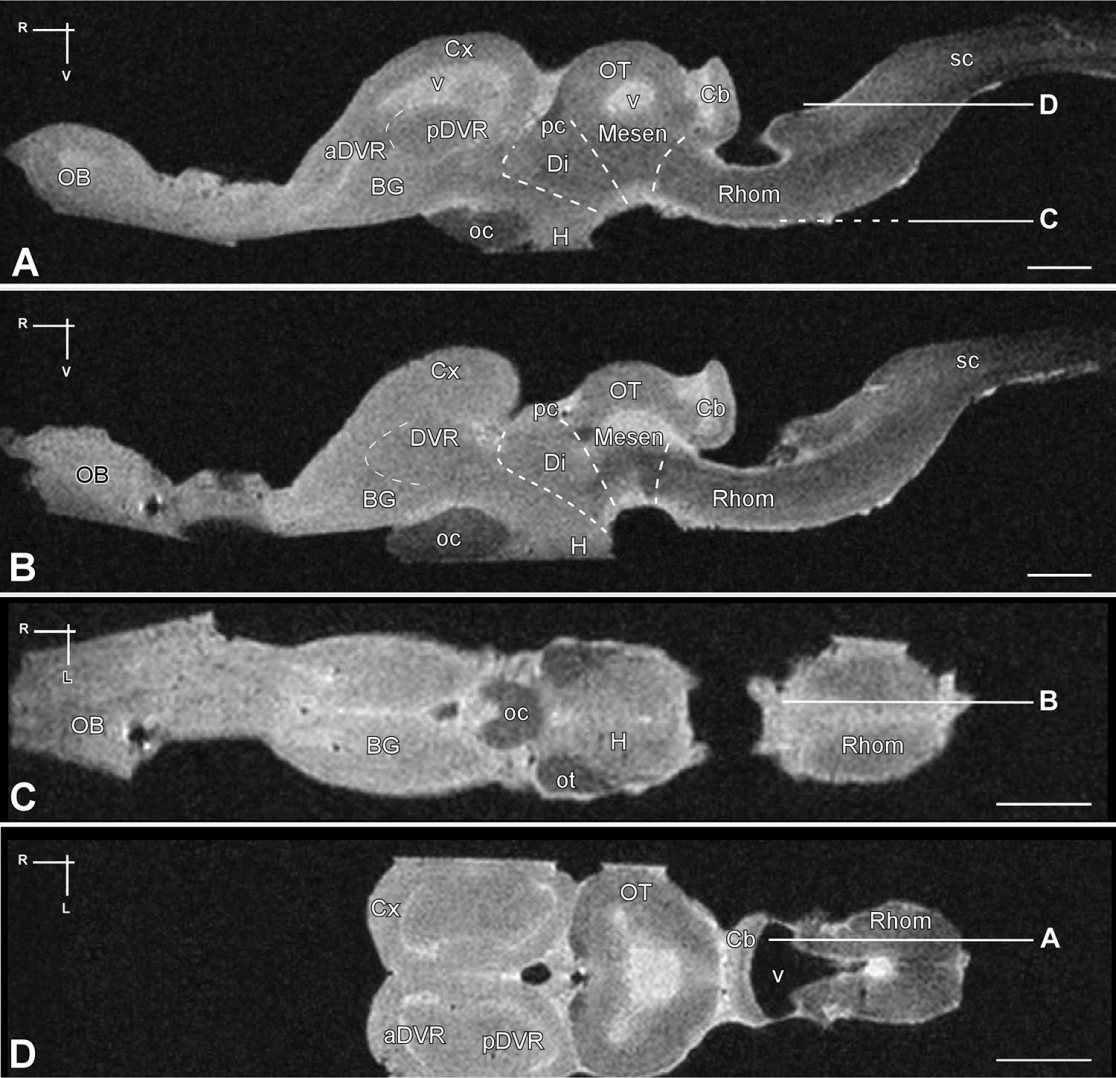
Sagittal and dorsal sections of the *Pseudopus apodus* brain from 3D MRI. Sagittal (**A**, **B**) and dorsal (**C**, **D**) sections of the *Pseudopus apodus* brain with the main brain regions indicated. Scale bar = 1 mm. See the list of abbreviations for reference

**Fig. 6:**
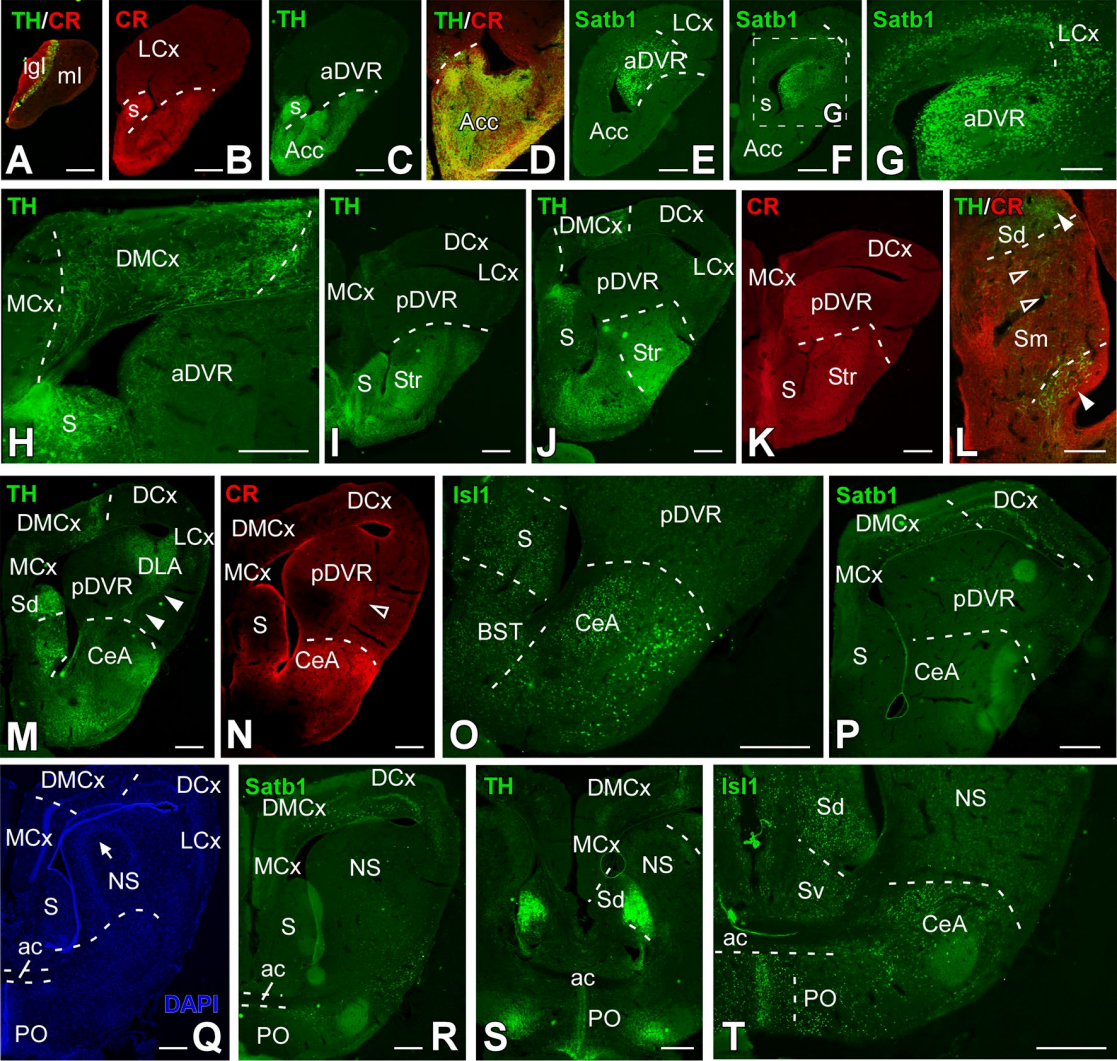
Transverse sections through the telencephalic areas of the *Pseudopus apodus* brain. Photomicrographs of transverse sections through the telencephalic areas of the *Pseudopus apodus* brain, showing the distribution of specific markers in particular nuclei. The color code for the markers is provided in each image. Scale bar A–C, E, F, I–K, M, N, P–R = 500 μm; D, G, H, L, O, S, T = 200 μm. See the list of abbreviations for reference

**Fig. 7:**
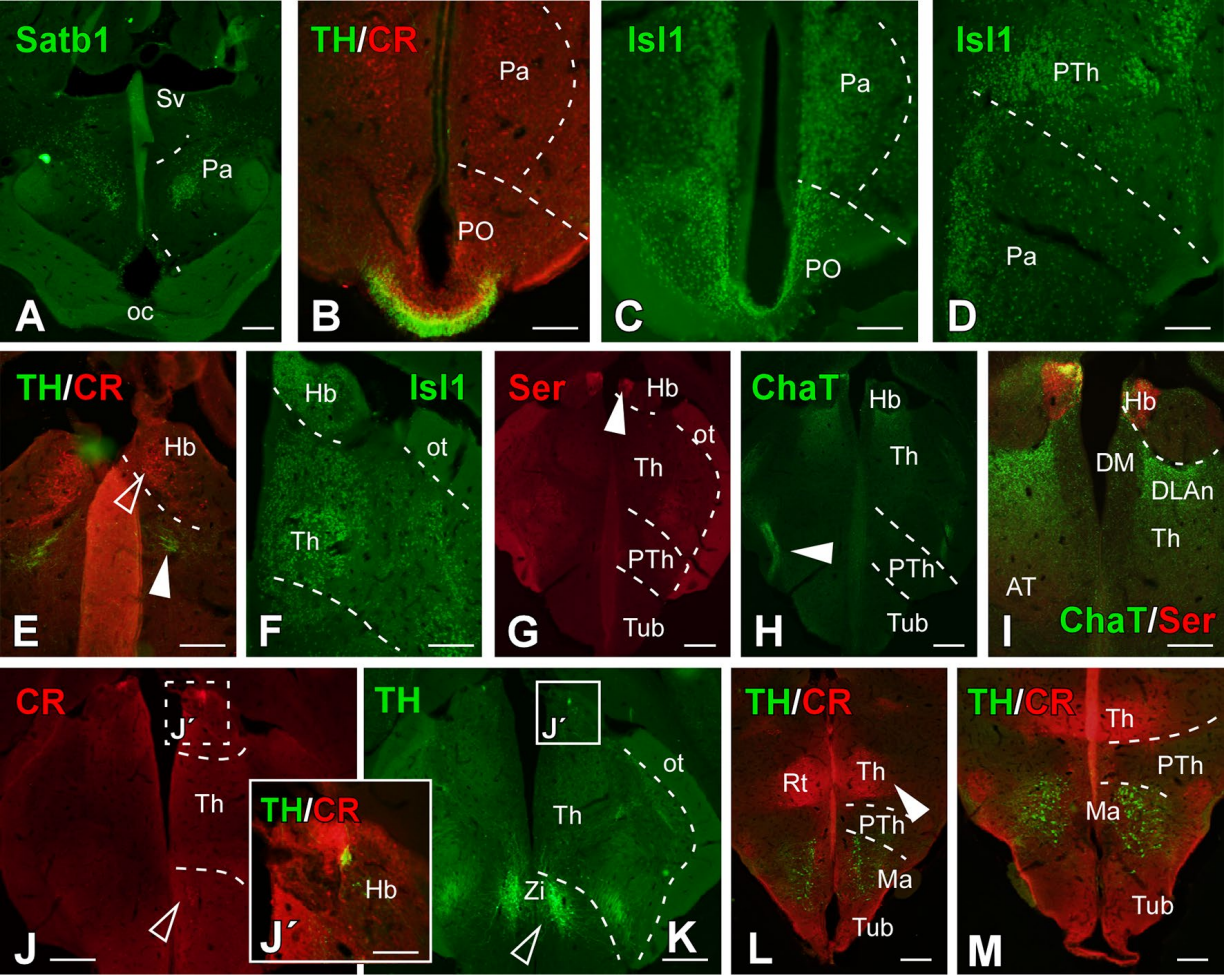
Transverse sections through hypothalamic and diencephalic areas of the *Pseudopus apodus* brain. Photomicrographs of transverse sections through hypothalamic and diencephalic areas of the *Pseudopus apodus* brain, showing the distribution of specific markers in particular nuclei. The color code for the markers is provided in each image. A–F, I–K = 200 μm; G, H, L, M = 500 μm. See the list of abbreviations for reference

**Fig. 8:**
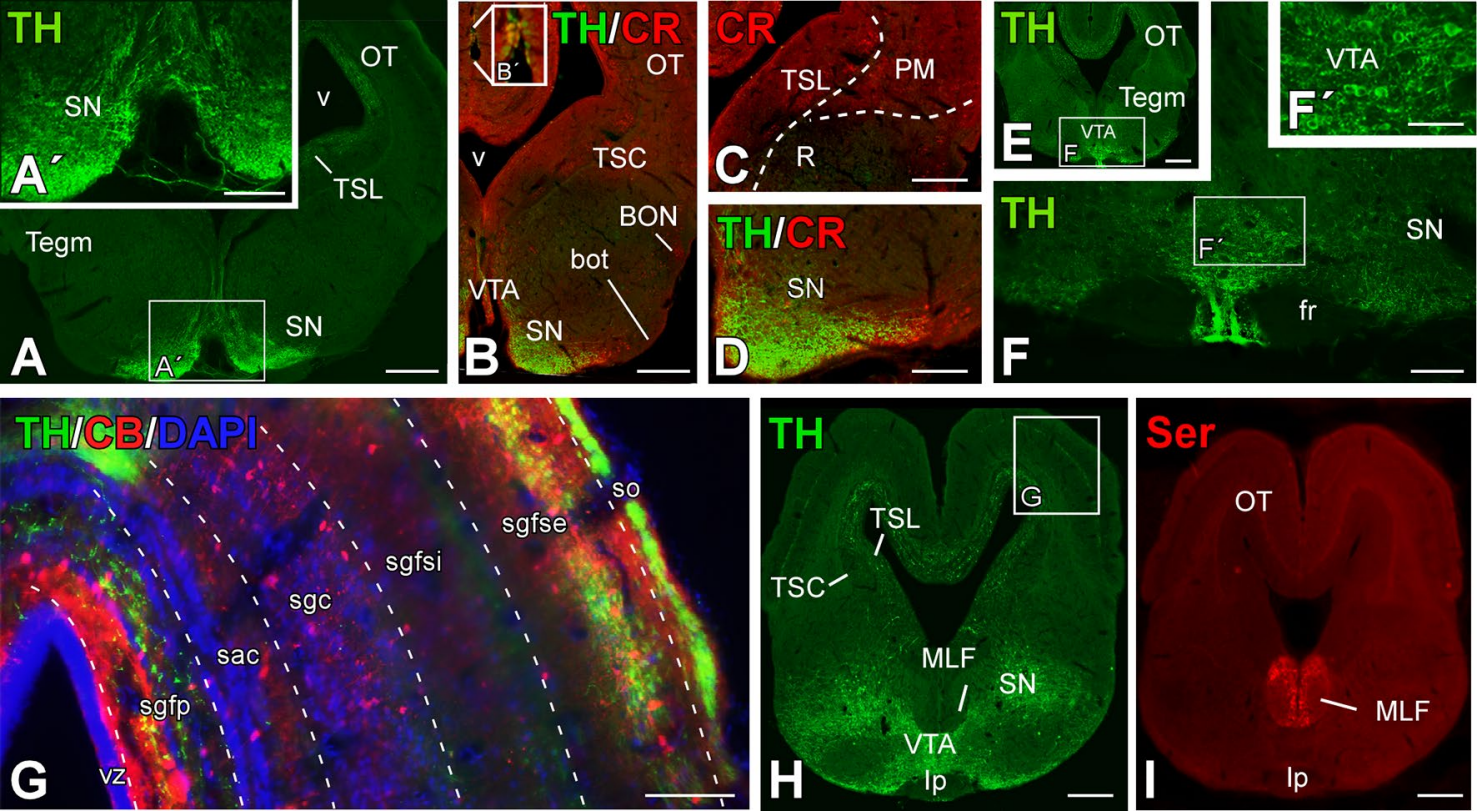
Transverse sections through mesencephalic areas of the *Pseudopus apodus* brain. Photomicrographs of transverse sections through mesencephalic areas of the *Pseudopus apodus* brain, showing the distribution of specific markers in particular nuclei. The color code for the markers is provided in each image. A, B, E, H, I = 500 μm; A′, C, D, F, G = 200 μm; F′ = 100 μm. See the list of abbreviations for reference

**Fig. 9:**
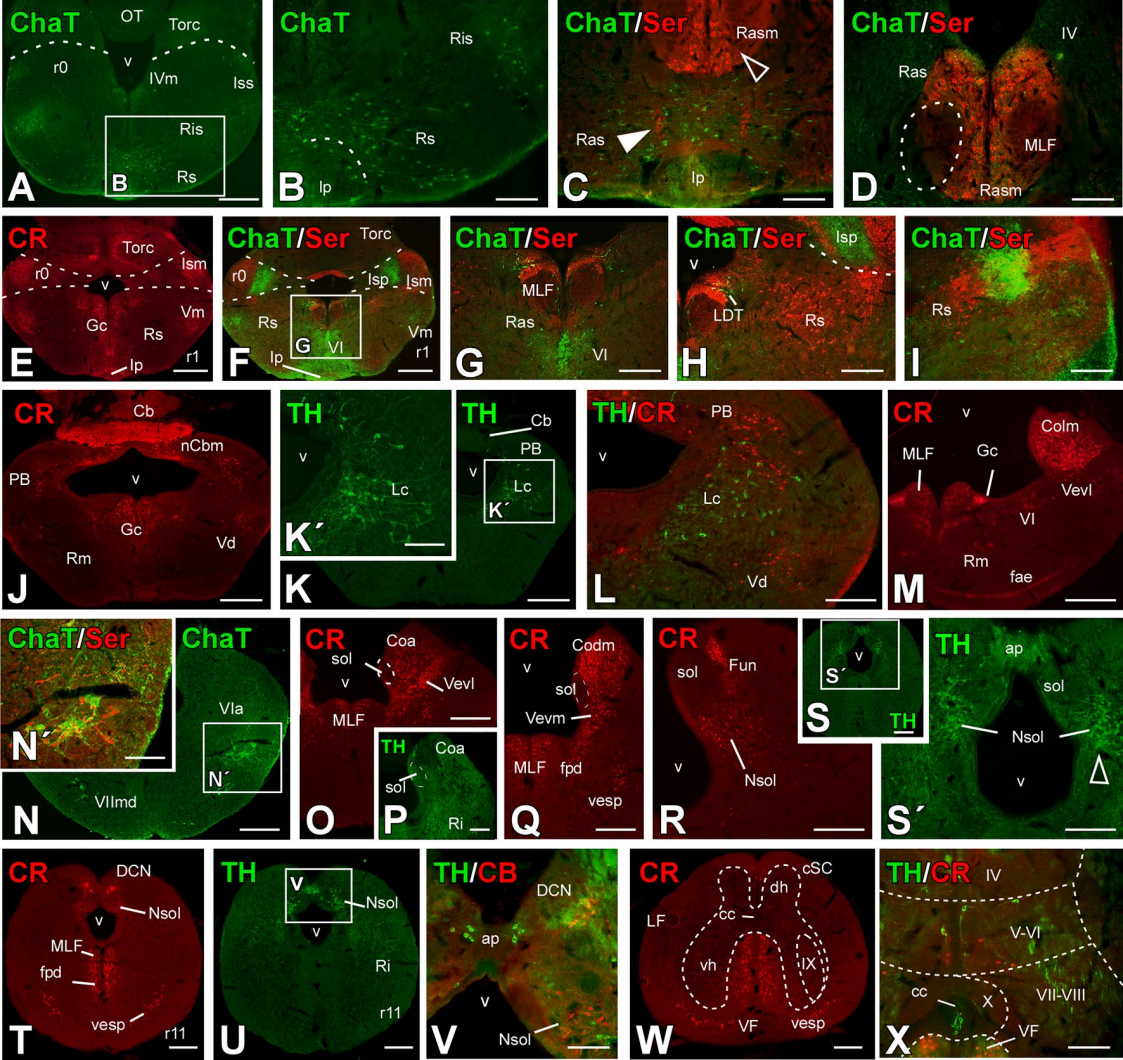
Transverse sections through Isthmic, rhombencephalic and rostral spinal cord areas of the *Pseudopus apodus* brain. Photomicrographs of transverse sections through Isthmic, rhombencephalic and rostral spinal cord areas of the *Pseudopus apodus* brain, showing the distribution of specific markers in particular nuclei. The color code for the markers is provided in each image. A, E, F, J, K, M, N, O, S, T, U, W = 500 μm; B–D, G-I, K′, L, N′, P, Q, R, S′, V, X = 200 μm. See the list of abbreviations for reference

At rostral levels of the telencephalon, using Nissl nuclear staining (Fig. 2A–I) the topographical dorsal region showed a layered organization known as the pallium. These layers form the cortical areas (medial: MCx, dorsomedial: DMCx, dorsal: DCx, and lateral: LCx, see Fig. 3A–C for a more detailed view), that cannot be identified through MRI (Fig. 4A–I). The most rostral levels of the pallial region coexists with a structure identified in lizards as the anterior dorsal ventricular ridge (aDVR). This nucleus, which lacks laminar organization, is adjacent to the laminated domain of the lateral cortex (Figs. 2B–D, 3A, B). This particular area in lizards is confined to anterior regions, but not in turtles (Jiménez et al. 2024). On the other hand, in the subpallial region, specifically at the most ventral tip, close to the lateral ventricle, another cellular clustering is observed, and due to its location, it is suggested to correspond to the accumbens nucleus (Acc; Fig. 2B, C). This nucleus is also visible through MRI (Fig. 4B, C). At the pallial levels where the aDVR disappears, it is substituted caudally by its most posterior part (pDVR). It represents the most prominent pallial structure in sauropsids (birds and reptiles), extending rostrocaudally (Fig. 2E, F). The pDVR is clearly identifiable through MRI in transverse sections (Fig. 4D-F), as well as in sagittal and dorsal sections (Fig. 5A, B, D). Further caudally, the position of the DVR is occupied by a structure organized in concentric layers, identified as the spherical nucleus (NS; Figs. 2G, H, 3C). Although the layers are not identifiable through MRI, the nucleus itself is visible (Fig. 4H, I). Ventrally at this level, in the subpallium, the basal ganglia (BG) are identified by Nissl staining (Fig. 2D–F) and by MRI (Fig. 4C–F). At the level of the anterior commissure (ac), in the caudal regions of the telencephalon, when the optic recess and optic chiasm (oc) become visible, the MRI allows a clear view of both tracts, as well as the extension of the oc (Figs. 2G, 4G), and the optic tract (ot; Figs. 2H, 3D, 4I). In the hypothalamic (H) and diencephalic regions, the high density of the cellular populations complicates the precise identification of specific nuclei without the use of specialized staining for these areas (compares Figs. 2H, I and 4H–L; Fig. 3D, E). However, MRI allows the precise recognition and mapping of the ventricular system's extent at these levels, which facilitates a clear identification of the anatomical territories. Caudally, the optic tectum (OT) in the mesencephalon is clearly visible, since it is highly developed and exhibits a well-defined laminar organization in this model (Figs. 2J–M, 3F). However, with the MRI technology used in this study, lamination in this region is only weakly suggested (Fig. 4L–P). In the mesencephalic tegmentum (Tegm), both nuclear staining (Figs. 2K–M, 3F′) and MRI (Fig. 4M–O) do not provide sufficient resolution to identify specific nuclei, although they allow for the identification of the major fiber tracts passing through this structure, similarly to what is observed in the rhombencephalic region (Figs. 2M–R, 4Q–Z). In the rostral rhombencephalon (Rhom), the cerebellum (Cb) is clearly identifiable (Figs. 2N, O, 4R, S), distinctly positioned, covering the fourth ventricle, as evidenced in the sagittal and dorsal MRI (Fig. 5A, B, D). This structure exhibits a heterogeneous organization in terms of cellular density, with a granular layer densely populated, clearly visible by MRI (Figs. 2O, 4S).To support this anatomical identification (Figs. 2, 3, 4 and 5), the expression patterns of key markers were analyzed using immunofluorescence (Figs. 6, 7, 8 and 9). These markers, previously described in the brains of other reptiles, allow a consistent identification of the regions outlined and their comparison with previously reported data.

At topographical more rostral levels, in the olfactory bulb (OB), well-organized layers are observed, containing CR-immunoreactive (-ir) cells that alternate with cells expressing TH (Fig. 6A). Caudally, both CR (Fig. 6B) and TH expression (Fig. 6C) persist in the rostral subpallium, which is essential for identifying the septum (S) and, therefore, delineating the boundary with the medial cortical region and ventrally with the Acc (Fig. 6D). At similar levels, in the pallial region, Satb1 expression (Fig. 6E–G) clearly defines the LCx, and aDVR, showing significant expression close to the ventricle. At more medial levels, the DMCx appears to have significant TH innervation, supporting its delineation (Fig. 6H). Furthermore, its subpallial innervation (Fig. 6I, J), along with CR expression (Fig. 6K), allows the identification of different subpallial populations, particularly in the medial region, where dorsoventral septal nuclei are identified with differential TH/CR expression, such as in the dorsal and medial portions (Sd; Sm; Fig. 6L). Further caudally, both expressions remain intense in the subpallium (Fig. 6M, N, S), as confirmed by comparison with Isl1 expression (Fig. 6O). At these levels, TH fibers also reach the region of the dorsolateral amygdala (DLA) closest to the ventricle (Fig. 6M). Similarly, the NS exhibits a notable TH innervation (Fig. 6S), although it does not show Satb1 expression (Fig. 6R). In the subpallium, TH staining clearly defines, at these levels, the dorsal portion of the septum (Sd; Fig. 6S), which is identified along with the ventral septum (Sv) and the BST and the central amygdala (CeA) by the expression of Isl1 (Fig. 6O, T). In Fig. 7, the specific expression of Satb1 defines the most anterior portion of the hypothalamus the paraventricular area (Pa) (Fig. 7A), distinct from the also positive telencephalic preoptic zone (PO) that in MRI images cannot be defined (Fig. 4A, G–I). Noticeably the optic chiasm in *Pseudopus* is massive and extends along preoptic and hypothalamic areas as seen in sagittal sections (Fig. 5) The anatomical regionalization of these two territories, telencephalic vs. hypothalamic, is achieved through the combination of TH and CR expression (Fig. 7B), and the additional expression of Isl1 further enables the identification of the hypothalamic-prethalamic boundary (Fig. 7C, D). The Isl1 pattern further supports the similarity with the Nissl-stained sections (Figs. 2H, 3E), demonstrating a peri/paraventricular hypothalamic organization. Caudally, the habenula is located in the dorsal portion (Fig. 7E–J). This structure shows CR-immunoreactive (CR-ir) cells in the dorsal part of the retroflex fasciculus, positive for TH (Fig. 7E, J), as well as Isl1-ir cells (Fig. 7F) and serotonergic innervation in the medial portion (see the arrowhead in Fig. 7G–I). The combination with cholinergic labeling (absent in this territory) confirms the localization of the medial portion of the habenula (Hb; Fig. 7G–I). In the thalamic region, serotonergic and TH innervation identify the hypothalamic-thalamic tract (Fig. 7G, K; previously observed by MRI, see Fig. 4J). Additionally, the zona incerta (ZI) is identified by intermingled CR-ir and TH-ir cells (see empty arrow in Fig. 7J, K). Finally, the intense CR expression in the thalamus, combined with TH, facilitates the identification of its boundary with the basal mammillary region of the hypothalamus (Ma, Fig. 7L, M). In the midbrain, the OT dorsally and the tegmentum (ventrally) are clearly identifiable by MRI, but other rostrocaudal or dorsoventral subdivisions are not observed. Immunohistochemical techniques revealed a clear alar region with the optic tectum that continued with the toral region that included the TH-ir zone corresponding to the torus laminaris (TSL; Fig. 8H), as well as CR-ir subpopulations of the torus semicircularis centralis (TSC) and the profundus mesencephali nucleus (PM). The basal tegmentum included a medial band that using TH labeling, revealed the most noticeable populations identified as the ventral tegmental area (VTA; Fig. 8E, F, H) and the substantia nigra (SN; Fig. 8A, B, D, H). The combination with CR-ir reveals additional distinct cell groups as the basal optic tract root nucleus (BON), and the red nucleus (R; Fig. 8C). Noticeably, the TH immunofluorescence combined with CB highlights the extent and distribution of the tectal layers (Fig. 8G), where fibers were marked in the periventricular gray layer (SGP) and the optic stratum (SO). Additionally, immunofluorescence with CB labeled both cells and fibers in the central gray stratum (sgc), the superficial external fibrous gray stratum (sgfs), and the stratum album centrale (sac). Caudally, at the isthmic region ChAT-ir cells were detected in the IVm nucleus and in the superficial isthmic nucleus (Iss), the isthmic reticular nucleus (Ris), and the interpeduncular nucleus (Ip; Fig. 9A, B). The isthmic complex is subdivided into a superficial portion (Iss; which is ChAT-ir), a medial 5HT-ir and CR portion (Ism; Fig. 9E, F) and a posterior portion (Isp; which is ChAT-ir; Fig. 9F). Serotonergic identification defined the raphe nuclei along caual part of the mesencephalon (Fig. 8I) and the hindbrain (Fig. 9C, D, F–I). Specifically, in the isthmic region, the combination with ChAT delineated large cells in the raphe superior nucleus (Ras), surrounding the medial longitudinal fasciculus (MLF), with significant cholinergic innervation both within the nucleus and in areas adjacent to the flm (Fig. 9D). Additional 5HT-ir cell bodies were found in a ventromedial position (Rasm; Fig. 9D). ChAT-ir cells were also identified in the interpeduncular nucleus (Ip) and fibers in the interpeduncular neuropil, originating from the retroflex fasciculus (fr; Fig. 9A–C). In the basal plate, motor neurons of the trigeminal nucleus (Vm) and the abducens nucleus (VIp) are labeled with ChAT-ir (Fig. 9F, G).

In the rhombomere 1, in the alar portion, Purkinje cells and the molecular layer of the Cb, along with the medial cerebellar nucleus (nCbm), are intensely labeled with CR-ir (Fig. 9J). Additionally, a population of CR-ir cells is observed in the corresponding parabrachial area (PB; Fig. 9J), where some TH-positive cells (Fig. 9K) are also detected. At this level, the locus coeruleus (LC) is identified by TH-ir (Fig. 9K, L). Caudally, CR-ir labels abundant fibers in the cochlear region, particularly in the lateromedial cochlear nucleus (Colm; Fig. 9M), the angularis cochlear nucleus (Coa; Fig. 9O), and the dorsomedial cochlear nucleus (Codm; Fig. 9Q). CR-ir cells are also observed in the lateroventral and medioventral vestibular nuclei (Vevl; Vevm; Fig. 9M, O, Q), as well as in the vestibulospinal tract (Vesp; Fig. 9Q, W) and the external arcuate fibers (fae; Fig. 9M). At caudal levels, separate parts of the solitary nucleus are labeled with CR and TH (Fig. 9R–V). Some TH-ir cells are found in the area postrema (ap), and CR-ir cells in the dorsal funiculus nucleus. Further caudally, in the Rhom (Fig. 9N–S), ChAT labeling identifies the ambiguous nucleus (A; Fig. 9N), which also expresses 5HT (Fig. 9N′).

Finally, in the rostral spinal cord (Fig. 9W, X), CR and TH labeling shows a population of cells in the dorsal horn, medially in laminae IV to VI, and a population of cells in contact with the cerebrospinal fluid (CSF) in TH-ir near the central canal (Fig. 9X). CR-ir fibers are observed in the ventral funiculus, originating from the vestibulospinal tract and the MLF.

## Discussion

In recent years, the use of magnetic resonance imaging (MRI) for neuroanatomical analysis has increased significantly (Lerch et al. 2017; Heuer et al. 2019; Friedrich et al. 2021). This growth can be attributed to several factors, including the reduction in the cost of MRI techniques and the recognition by the neuroscience community of the importance of a broader approach to neurobiological modelling, enhancing studies of the evolution of the nervous system. The inclusion of a wide range of models expands the scope of knowledge, but at the same time requires careful analysis to establish new neuroanatomical and evolutionary frameworks for future research. This context has undoubtedly led to significant advances but also presents considerable challenges.

This is the primary context of our study, which provides a comprehensive neuroanatomical investigation of the brain of the legless lizard *Pseudopus apodus* using a combination of MRI, Nissl staining, and specific immunofluorescence detection of conserved markers. This research is particularly novel in that it explores a model that has been used infrequently in neuroanatomical studies, in addition, the evolutionary significance of this research is noticeable, as *Pseudopus apodus*, a legless lizard, offers an exceptional model from an evolutive and comparative ecological perspective. Since the habitat preferences of *Pseudopus apodus* are diverse, reflecting its adaptability to varying geographical and environmental conditions. This species also demonstrates significant morphological plasticity, even across small geographical distances, suggesting that environmental factors such as temperature, precipitation, and predatory fauna may influence its morphological traits (Glavaš et al. 2020). This is consistent with other species exhibiting phenotypic plasticity, such as *Podarcis siculus* (Herrel et al. 2008). Thus, this model offers new insights into the organization of the lizard brain and provides a comparative analysis with existing descriptions of reptile neuroanatomy. In addition, as advances are made in the use of magnetic resonance imaging (MRI) techniques and histological analyses to study lesser-researched species, new opportunities are opening up to question established evolutionary paradigms. In this case, it is interesting to consider whether the unique neuroanatomical features of *Pseudopus apodus* constitute a window into the evolution of the neurobiology of legless reptiles, considering the morphological plasticity observed in the species.

Comparative analyses of *Pseudopus apodus* have previously focused on its anatomy, including detailed examinations of the pectoral and pelvic girdles and the hindlimbs. These studies have shown that *Pseudopus apodus* exhibits anatomical similarities with *Ophisaurus* species from North America, North Africa, and Southeast Asia (Klembara et al. 2022). Furthermore, its autapomorphic skull features clearly distinguish it from both *Anguis* and *Ophisaurus* (Klembara et al. 2017), a distinction further supported by molecular data (Jandzik et al. 2018).

In a recently published study we employed a similar methodology presenting a comparative atlas of reptilian brains, including both lizards and snakes, analyzed using MRI and nuclear labeling techniques (Jiménez et al. 2024). The data from that study, along with the findings from the current research, will be essential for distinguishing which cerebral traits are intrinsic to lizards and which are specific to snakes, independent of the functional adaptations associated with limb-lessness. Within the Anguidae family, varying degrees of limb reduction can be observed, ranging from partial reduction to complete limb-lessness. While the locomotor patterns of limbless anguilliform lizards are like those of snakes, they exhibit less developed ventral scales. In lizards, these scales play a crucial role in facilitating efficient tail-based slide-pushing locomotion, generating high lateral friction and propulsion (Spinner et al. 2015). These features underscore important distinctions between limbless anguids and snakes, highlighting their divergence from a truly tailless condition. Similarly, the brain of *Pseudopus apodus* resembles that of other lizards, with species-specific characteristics, but does not exhibit the same structural traits as those found in snakes (Jiménez et al. 2024).

### MRI vs classical neuroanatomy: complementary insights and utilities in brain anatomy analysis

The application of non-invasive techniques to species that are rarely studied in research but of great scientific interest deserves further consideration, both for their usefulness to animal conservation organizations (such as zoos or wildlife rehabilitation centers) and for the valuable information they provide for comparative and evolutionary studies, providing new information for the study of neuroanatomy-function-ecology relationships. It is therefore important to determine which regions of the brain can be reliably imaged and analyzed by MRI. In addition, it also identifies at what detail or resolution they can be analyzed, as for example, MRI facilitates the visualization of large-scale brain structures, such as the dorsal ventricular crest and the nucleus accumbens, while histological analysis provides a high-resolution view of finer cellular details, such as the laminar organization of the dorsal cortex. This complementary approach highlights the inherent strengths and limitations of each technique: MRI allows observation of macroscopic structures but lacks the resolution needed to discern architecture at the cellular level, while histological techniques provide fine cellular detail but are limited by their inability to visualize large structures non-invasively.

In the rostral portion of the brain, especially in the pallium, MRI and histological analysis reveal well-organized regions, such as the dorsal and lateral cortex, although, likewise, the DVR, a key structure in reptiles involved in the processing of visual and auditory information, extends rostrocaudally and is visualized both in tissue sections and by MRI.

Regarding the regions derived from the lateral pallium in reptiles, it is important to highlight the structural differences among various taxa. Turtles exhibit a prominent nucleus known as the pallial thickening (PT), a well-defined structure that appears to be absent in lizards or reduced to only rostral regions (Jiménez et al. 2024; Desfilis et al. 2018). From a genetic and functional perspective, the PT of turtles shows similarities to the anterior region of the dorsal ventricular ridge (aDVR) in *Pogona vitticeps* and to the claustrum in mammals (Schede et al. 2021; Norimoto et al. 2020; Tosches et al. 2018). Our MRI data in *P. apodus* do not allow for the identification of a distinct nucleus equivalent to the PT, in contrast to what has been observed in previous MRI studies of turtles (Jiménez et al. 2024). This finding suggests that the PT in turtles may be homologous to the aDVR of *P. apodus*, a region that we have identified through Satb1 expression, similar to what has been reported in other lizards such as *P. vitticeps* (Tosches et al. 2018) or *P. picta* (Rueda-Alaña et al. 2025), and turtles (*own results*). Further studies in *P. apodus* are necessary to determine whether this region is also involved in sleep regulation, as observed in other lizards, as well as in visual processing. The latter aspect is particularly relevant, given that the PT in turtles receives visual input from the dorsal lateral geniculate nucleus (Hall and Ebner 1970).

On the other hand, the most posterior region of the DVR (pDVR) has been identified as a multisensory integration center functionally similar to particular nuclei of the pallial amygdala in mammals. This identification is based on both its hodological characteristics (Martínez-García and Lanuza 2009) and its cellular composition (Desfilis et al. 2018; Tosches et al. 2018) and is associated with emotional behaviors such as fear (Davies et al. 2002). In this regard, we observed in *P. apodus* a TH-immunoreactive innervation extending from the basal ganglia to the lateralmost region of the pDVR, reaching the ventricle. These amygdalostriatal projections have been previously described in reptiles (Novejarque et al. 2004). Our findings in *P. apodus* further support the hypothesis that the pDVR constitutes part of the pallial reptilian amygdala, as the presence of TH has been demonstrated in both the striatum and the developing amygdala of mammals (Bupesh et al. 2014). These data are relevant as they contribute to understanding the role of catecholaminergic neurons, whose presence in reptiles suggests their key importance in the evolution of the modulation of emotional behaviors.

Squamates, including lizards and snakes, possess a sophisticated vomeronasal chemical detection system (Schwenk 1994; Cooper Jr 1995, 1996; Filoramo and Schwenk 2009). Both groups use their tongue to sample environmental cues, transporting these chemical signals to the vomeronasal complex, located anatomically above it (Filoramo and Schwenk 2009). Beyond this common function, however, the vomeronasal system exhibits considerable variation across species, with evolutionary implications (Baeckens et al. 2017). It has been suggested that snakes exhibit the most elaborate and refined behavioral adaptations related to this sensory anatomy (Schwenk 1994). In contrast, simpler models such as iguanas possess fleshy tongues with reduced chemoreceptive capacity [*see discussion in* (Zhan et al. 2024)]. This raises an intriguing question: based on the anatomy of the tongue and vomeronasal organ (Graves and Halpern 1990; Halpern and Kubie 1983; Halpern 2007), can we infer the architecture of brain centers involved in processing these sensory inputs? and perhaps even make predictive models. The nucleus sphericus (NS), a primary secondary vomeronasal area in the squamate telencephalon (Lanuza and Halpern 1997; Martínez-Marcos et al. 2002; Martínez-Marcos and Halpern 2009), provides an ideal case for such analysis. Comparative MRI analysis is thus a valuable tool in evolutionary studies, as demonstrated in reptiles (Jiménez et al. 2024) and in *Pseudopodus* in this study. The NS is easily identifiable by MRI, allowing for the exploration of the relationships between the tongue, the vomeronasal complex, and the NS.

The diencephalic, hypothalamic, mesencephalic, and rhombencephalic regions present particular challenges for both MRI and histological analysis due to the density and complexity of the nuclei in these areas. The intricate organization of these regions complicates the distiction of specific nuclei without the use of specialized markers. However, in this respect, MRI allows a clear identification of structures such as the optic tectum, and the cerebellum, which are undoubtedly two very powerful tools of analysis at an evolutionary level. First of all, at the macro level, the anatomy of both regions in the case of *Pseudopus* is comparable to that described in other models of lizards, rather than snakes, even though they share a legless condition in both cases (ten Donkelaar 1998). And in terms of domains, the results obtained also demonstrate the conserved condition of this species with respect to other lizards, supporting that, at least in this case, the conditions of locomotor adaptation are likely to be observed at three levels, which opens an interesting question for example in the analysis of the spinal cord. Finally, MRI is effective in tracing the expansion of the ventricular system, offering important insights despite the limitations of non-specific staining and the resolution constraints of MRI in these densely populated brain regions. These findings highlight the need for further studies employing targeted marker-based approaches to achieve a more precise understanding of these complex regions.

### Previous neuroanatomical studies on this model

The use of markers, neurotransmitters, neuropeptides and, in recent years, gene expression patterns as tools to define, confirm, regions and boundaries is very useful in neuroantomical studies (Puelles and Ferran 2012). The expression of neurotransmitters in the lizard brain has been analyzed in detail in recent years, although the scarcity of gene expression data in reptiles is still high. And in this context, in the case of *P. apodus*, the lack or even absence in the case of genoarchitectural analysis is total. Taking this into account, the markers used in this study, both neurotransmitters and calcium-binding proteins, as well as transcription factors, are markers widely described in other reptiles and that have been useful for the analysis of populations and/or regions and specific limits (Pierre et al. 1990; Medina et al. 1993, 1994; Guirado et al. 1999a, b; Smeets et al. 2006, 2001; Báez et al. 2003; Morona et al. 2006; Yan et al. 2010; Moreno et al. 2010, 2012; Domínguez et al. 2015; Tosches et al. 2018; Desfilis et al. 2018; Wang et al. 2021b; Hain et al. 2022; Rueda-Alaña et al. 2025). Thus, the existing neuroanatomical research on this model remains limited, with most studies dating back several decades and, in many cases, being largely inaccessible. However, extensive research has been conducted on telencephalic connectivity in reptiles, particularly lizards (Martínez-Garcia and Lanuza 2009; Lanuza et al. 2002; Hall 2008; Guirado and Dávila 2002; Bruce and Butler 1984a, b), providing much evidence for comparison. For example, connections between the septum and the medial cortex (MCx) have been documented (Belekhova and Nemova 1988), showing similarities to the hippocampal-septal connections found in all studied tetrapods (González and López 2002). Additionally, the MCx connectivity with the mammillary complex in these lizards resembles to that of mammals, supporting the idea of a homologous function (Belekhova and Kenigfest 1983). Notably, early classical anatomical studies of the medial cortex in this model established the involvement of the hippocampal-like cortex in conditioned alimentary reflexes and the dorsal ventricular ridge in the visual processing (Ivazov 1983), suggesting a high degree of evolutionary conservation of these brain structures. Sensory input is known to reach the MCx via the medial forebrain bundle and the anterior thalamus (Belekhova and Ivazov 1983) and notably, the thalamus also exhibits conserved features, such as GABA expression in the dorsal lateral geniculate nucleus (Rio et al. 1992) and the established connections between the ventral lateral geniculate nucleus and the hypothalamus (Belekhova 1991). Additionally, TH-positive innervations can be observed in the dorsomedial cortex of *P. apodus*, similar to what occurs in the cornu ammonis 3 (CA3) region of mammals (Milner and Bacon 1989). This finding provides further evidence of the homology between mammal-hippocampus and reptile-MCx/DMCx (Reiter et al. 2017).

The neurochemistry of the lizard brain has been extensively explored, leading to the identification of its primary subdivisions and enabling evolutive comparisons with other reptiles and amniotes (Medina et al. 1993; Báez et al. 2003; Tosches et al. 2018; Desfilis et al. 2018; Hain et al. 2022; Wang et al. 2021a). However, research focusing specifically on apoda lizards remains relatively scarce. The analysis of specific markers, such as Satb1, CR, 5HT and TH has significantly advanced our understanding of the brain regions. For instance, high levels of Satb1 expression in the anterior dorsal ventricular ridge (aDVR) allow for clear delineation of this rostral region from the more caudal part of the DVR, which does not exhibit Satb1 expression. Similarly, distinct staining patterns in the nucleus accumbens, dorsal cortex, and thalamic regions contribute to their anatomical identification. Furthermore, serotonin expression in the brain of this model closely mirrors that observed in other lizards (Pierre et al. 1990). In summary, the application of these markers not only corroborates previous findings in reptilian neuroanatomy but also offers novel insights into the organization of brain regions in *Pseudopus apodus*, contributing to a more refined understanding of its neural architecture.

## Data Availability

The rows of all models are not publicly available due to privacy reasons but are available upon request to the corresponding author.

## Abbreviations

Acc: Nucleus accumbens
ac: Anterior commissure
aDVR: Anterior part of the DVR
ap: Area postrema
AT: Area triangularis
BG: Basal ganglia
BON: Basal optic nucleus
bot: Basal optic tract
BST: Bed nucleus of the stria terminalis
Cb: Cerebellum
cc: Central canal
CeA: Central amygdala
Colm: Coclear lateromedial nucleus
Coa: Coclear angularis nucleus
Codm: Coclear dorsomedial nucleus
cSC: Cervical levels of the spinal cord
Cx: Cortex
DCN: Dorsal column nucleus
DCx: Dorsal cortex
Di: Diencephalon
DMCx: Dorsomedial cortex
DM: Dorsomedial nucleus
DLA: Dorsolateral amygdala
DLAn: Dorsolateral anterior nucleus
DVR: Dorsal ventricular ridge
fae: External arcuate fibers
fpd: Fasciculus predorsalis
fr: Fasciculus retroflexus
Gc: Central grey
Hb: Habenula
H: Hypothalamus
IIIv: Third ventricle
Igl: Internal granular layer
Ip: Interpeduncular neuropil
Ipn: Interpeduncular nucleus
Is: Isthmus
Iss: Superficial isthmic nucleus
Isp: Posterior isthmic nucleus
Ism: Medial isthmic nucleus
IV: Troclear nucleus
IVm: Motor troclear nucleus
IVv: Fourth ventricle
IX: Lamina of the spinal cord
LCx: Lateral cortex
Lc: Locus ceruleus
LDT: Laterodorsal tegmental nucleus
LF: Lateral funiculus of the spinal cord
Ma: Mammillary area
MCx: Medial cortex
Mesen: Mesencephalon
ml: Mitral cell layer of the olfactory bulb
MLF: Medial longitudinal fasciculus
Nsol: Nucleus of the solitary tract
NS: Nucleus sphericus
nCbm: Nucleus cerebellosus medialis
OB: Olfactory bulb
oc: Optic chiasm
on: Optic nerve
OT: Optic tectum
ot: Optic tract
Pa: Paraventricular nucleus of hypothalamus
PB: Parabrachial area
pc: Posterior commissure
pDVR: Posterior part of the DVR
pi: Pineal gland
PM: Profound mesencephalic area
PO: Preoptic area
PTc: Pretectum
PTh: Prethalamus
R: Red nucleus
r0: Isthmus
r1–r11: Rhombomeres 1 to 11
Ras: Raphe superior
Rasm: Raphe superior medial
Rhom: Rhombencephalon
Ri: Inferior reticular nucleus
Ris: Istmic reticular nucleus
Rm: Median reticular nucleus
Rt: Nucleus rotundus
S: Septum
sac: Stratum album centrale
sc: Spinal cord
SC: Suprachiasmatic nucleus
Sd: Dorsal septum
sgc: Stratum griseum centrale
sgfp: Stratum griseum et fibrosum periventriculare
sgfsi: Inner part of stratum griseum fibrosum et superficiale
sgfse: External part of stratum griseum fibrosum et superficiale
SN: Substantia nigra
Sm: Medial septum
so: Stratum opticum
sol: Solitary tract
Str: Striatum
Sv: Ventral septum
Tegm: Tegmentum
Tel: Telencephalon
Th: Thalamus
TSC: Central nucleus of the torus semicircularis
TSL: Laminar nucleus of the torus semicircularis
Tub: Tuberal area of the hypothalamus
Tor: Torus
Torc: Torus caudalis
v: Ventricle
Vd: Descending nucleus of the trigeminal tract
vesp: Trigeminoespinal tract
Vevl: Nucleus vestibularis ventrolateralis
Vevm: Nucleus vestibularis ventromedialis
Vllmd: Facial nucleus medio-dorsal part
Vl: Trochear nucleus
VF: Ventral funiculus
vh: Ventral horn of the spinal cord
Vm: Trigeminal motor nucleus
VTA: Ventral tegmental area
V-VI: Layers of the spinal cord
vz: Ventricular zone
X: Periependimary layer of the spinal cord
VII-VIII: Layers of the spinal cord
Zi: Zona incerta
